# Retraction: Towards robot-assisted therapy for children with autism—the ontological knowledge models and reinforcement learning-based algorithms

**DOI:** 10.3389/frobt.2026.1873855

**Published:** 2026-05-15

**Authors:** Frontiers Editorial Office

The journal retracts the 06 April 2022 article cited above.

Following publication, concerns were raised regarding the dataset used in this article. The dataset is described as containing images of children with autism spectrum disorder (ASD) however, the images appear to have been gathered from the internet without documented clinical histories or confirmation of an ASD diagnosis. There is also no documented evidence of ethical oversight or consent from the children or from their parents or legal guardians.

As a result of these concerns, and in line with Frontiers’ policies and those of COPE, the publisher no longer has confidence on the results and conclusions of this article. To protect the participants, any potentially identifiable data have been removed from the article.

This retraction was approved by the Chief Editors of Frontiers in Robotics and AI and the Chief Executive Editor of Frontiers. The authors agree to this retraction.

